# Supplementary material for: Three types of remapping with linear decoders: a population-geometric perspective
Source: bioRxiv. 2025 Aug 11:2025.03.14.643251. Preprint. [Version 2] doi: 10.1101/2025.03.14.643251 (PMC12363822; doi:10.1101/2025.03.14.643251)
Supplement: Supplement 1 [file NIHPP2025.03.14.643251v2-supplement-1.pdf]

bioRxiv preprint doi: https://doi.org/10.1101/2025.03.14.643251; this version posted August 11, 2025. The copyright holder for this preprint (which was not certified by peer review) is the author/funder, who has granted bioRxiv a license to display the preprint in perpetuity. It is made available under aCC-BY 4.0 International license.

Supporting Information

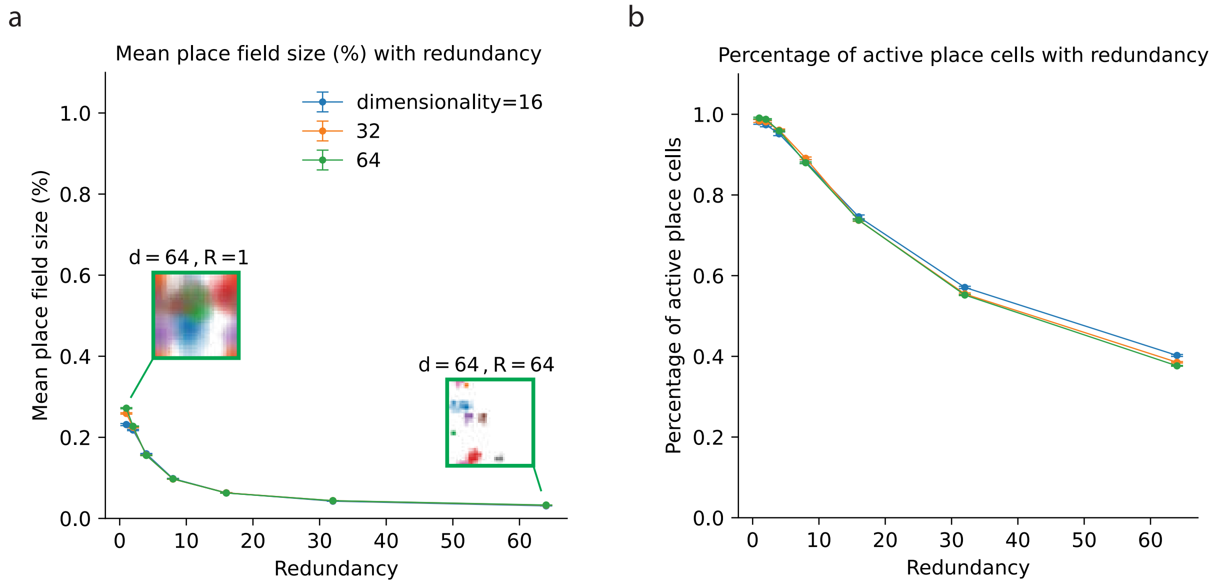

Figure S1: **Place cell statistics for a single environment**
a,b: Mean place field size (as % of the environment; **a**) and percentage of active place cells in each environment (**b**) as a function of redundancy (N/Y) for three different dimensionalities (Y) for multi-chart ED (mean across neurons and 10 environments, with SEM across environments). Example place fields shown in (**a**) plotted for (Y, redundancy) = (64, 1) and (64, 64). Related to the distribution of percentage of neurons active in n rooms (see Fig. S3c).

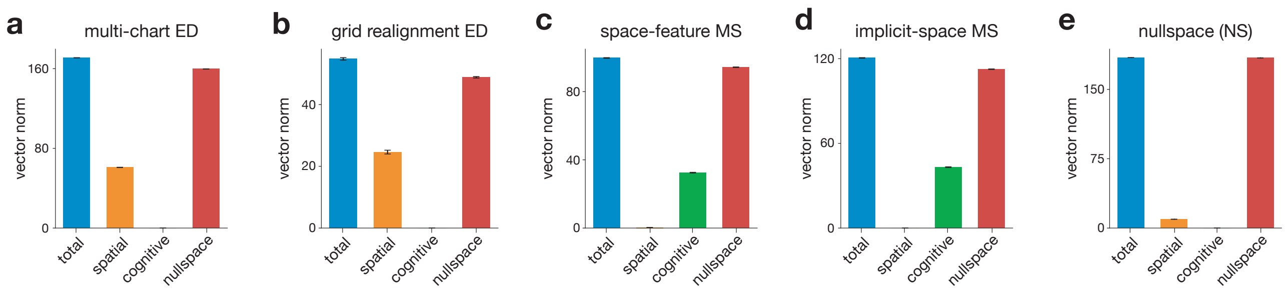

Figure S2: **Comparison of remapping changes in each component of pseudo-linear encoder.**
Norm of the remapping vector between 10 environments computed as total ($||\mathbf{r}^A - \mathbf{r}^B||$); spatial ($||\mathbf{D_p r}^A - \mathbf{D_p r}^B||$, which is equal to $||\mathbf{y}^A - \mathbf{y}^B|| = ||(\mathbf{R}^A - \mathbf{R}^B)\mathbf{z_p}||$ for ED and NS remapping and $||\mathbf{z_p}^A - \mathbf{z_p}^B||$ for MS remapping); cognitive ($||\mathbf{D_c r}^A - \mathbf{D_c r}^B|| = ||\mathbf{z_c}^A - \mathbf{z_c}^B||$); and NS ($||(\mathbf{r}^A - \mathbf{r}^B) - \mathbf{E}((\mathbf{z}^A - \mathbf{z}^B)|| = ||\boldsymbol{\nu}^A - \boldsymbol{\nu}^B||$). Shown for main figure examples of multi-chart ED remapping (**a**), grid realignment ED remapping (**b**), space-feature MS remapping (**c**), implicit-space MS remapping (**d**), and NS remapping (**e**).

bioRxiv preprint doi: https://doi.org/10.1101/2025.03.14.643251; this version posted August 11, 2025. The copyright holder for this preprint (which was not certified by peer review) is the author/funder, who has granted bioRxiv a license to display the preprint in perpetuity. It is made available under aCC-BY 4.0 International license.

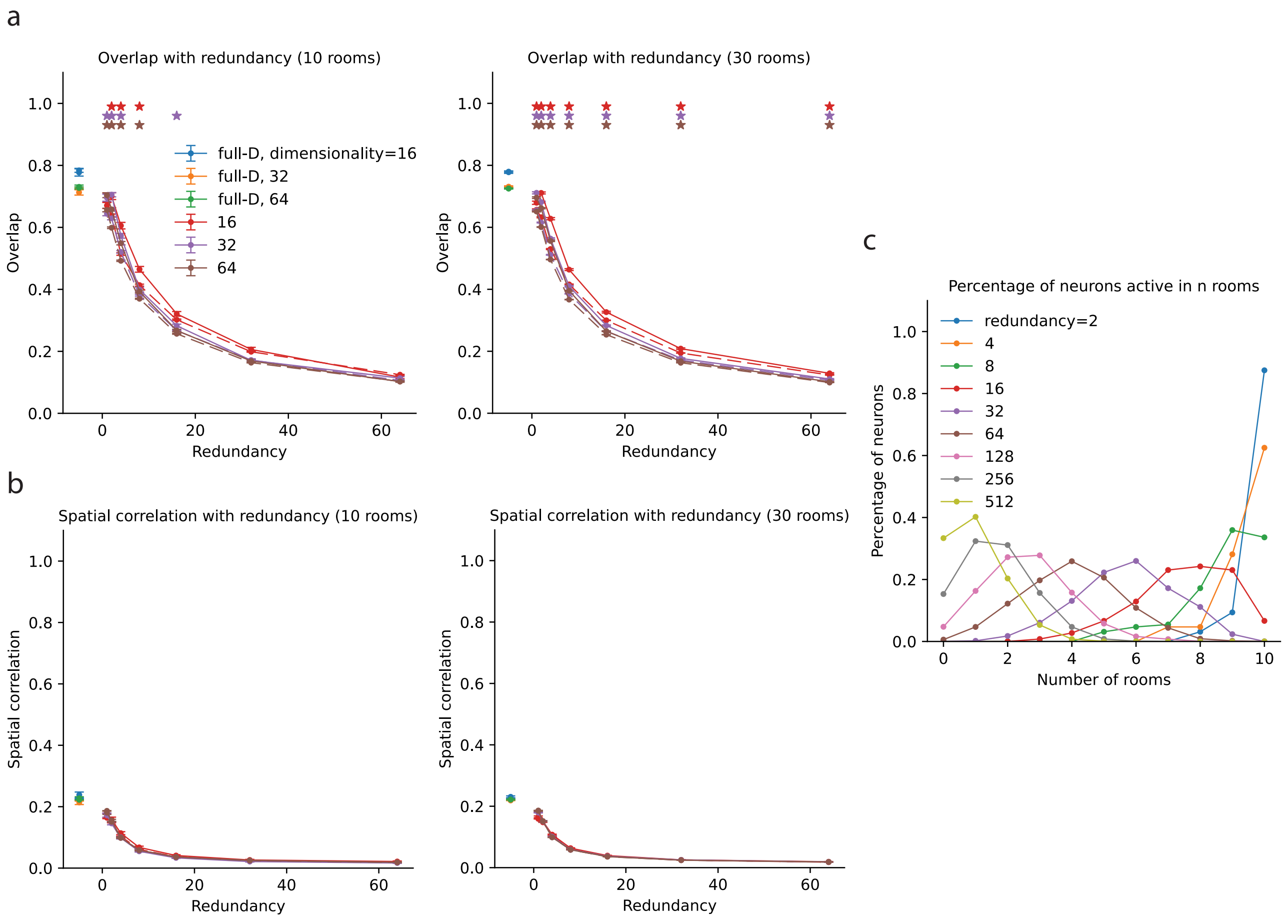

Figure S3: **Multi-chart encoder-decoder (ED) remapping analysis.**
a: Overlap (solid) and shuffle overlap (dashed) between 10 (left) and 30 (right) environments as a function of redundancy (N/Y) for different dimensionality (Y) values. Stars mark where the mean overlap is significantly different from the shuffle mean (t-test, Bonferroni correction with $n = 3$ for full-D and $n = 21$ for low-D, see Methods Section 4). The full-dimensional embedding case ($N/Y = 1$; Methods Section 2.1.1; Fig. S4) is plotted at an x-axis value of -1 to differentiate it from the other cases (blue, orange, green). Note the difference significance levels for left versus right by changing the amount of data (10 versus 30 environments). **b**: Spatial correlation, same as in (a). Note that none of the data is significantly different from random in this case. **c**: Histogram of percentage of neurons active in n rooms for different redundancies, measured over 10 rooms as in left panels in (**a**, **b**).

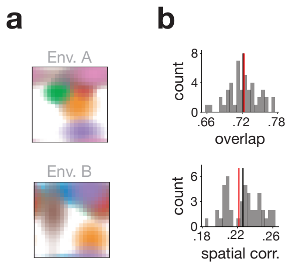

Figure S4: **Multi-chart encoder-decoder (ED) remapping with full-dimensional (full-D) embedding space.** **a**: Example place field rate maps for two environments. **b**: Overlap and spatial correlation distributions for 10 rooms, with mean (black) and comparison with a shuffle control (red), showing consistency with truly random remapping. Related to full-D simulations (plotted at x-axis value of -1) from Fig. S3a,b.

bioRxiv preprint doi: https://doi.org/10.1101/2025.03.14.643251; this version posted August 11, 2025. The copyright holder for this preprint (which was not certified by peer review) is the author/funder, who has granted bioRxiv a license to display the preprint in perpetuity. It is made available under aCC-BY 4.0 International license.

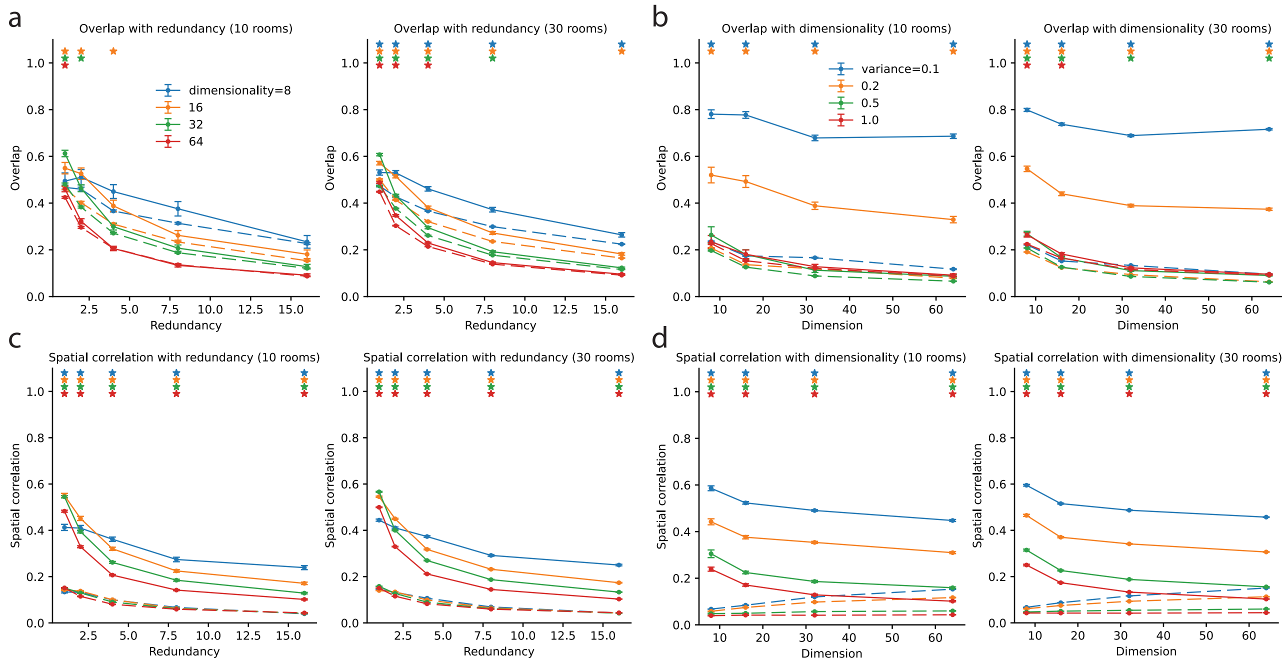

Figure S5: **Space-feature mixed-selective (MS) remapping analysis.**
a: Overlap (solid) and shuffle overlap (dashed) between 10 (left) and 30 (right) environments as a function of redundancy (N/Y) for different dimensionality (Y) values. Stars mark where the mean overlap is significantly different from the shuffle mean (t-test, Bonferroni correction with n = 20, see Methods Section 4). **b**: Overlap (solid) and shuffle overlap (dashed) between 10 (left) and 30 (right) environments as a function of dimensionality (Y) for different values of cognitive variables variance (σ, see Methods Section 2.2.1). Stars mark where the mean overlap is significantly different from the shuffle mean (t-test, Bonferroni correction with n = 20, see Methods Section 4). Note the difference significance levels for left versus right in panels (**a**, **b**) by changing the amount of data (10 versus 30 environments). **c**: Spatial correlation, same as in (a). **d**: Spatial correlation same as in (b).

bioRxiv preprint doi: https://doi.org/10.1101/2025.03.14.643251; this version posted August 11, 2025. The copyright holder for this preprint (which was not certified by peer review) is the author/funder, who has granted bioRxiv a license to display the preprint in perpetuity. It is made available under aCC-BY 4.0 International license.

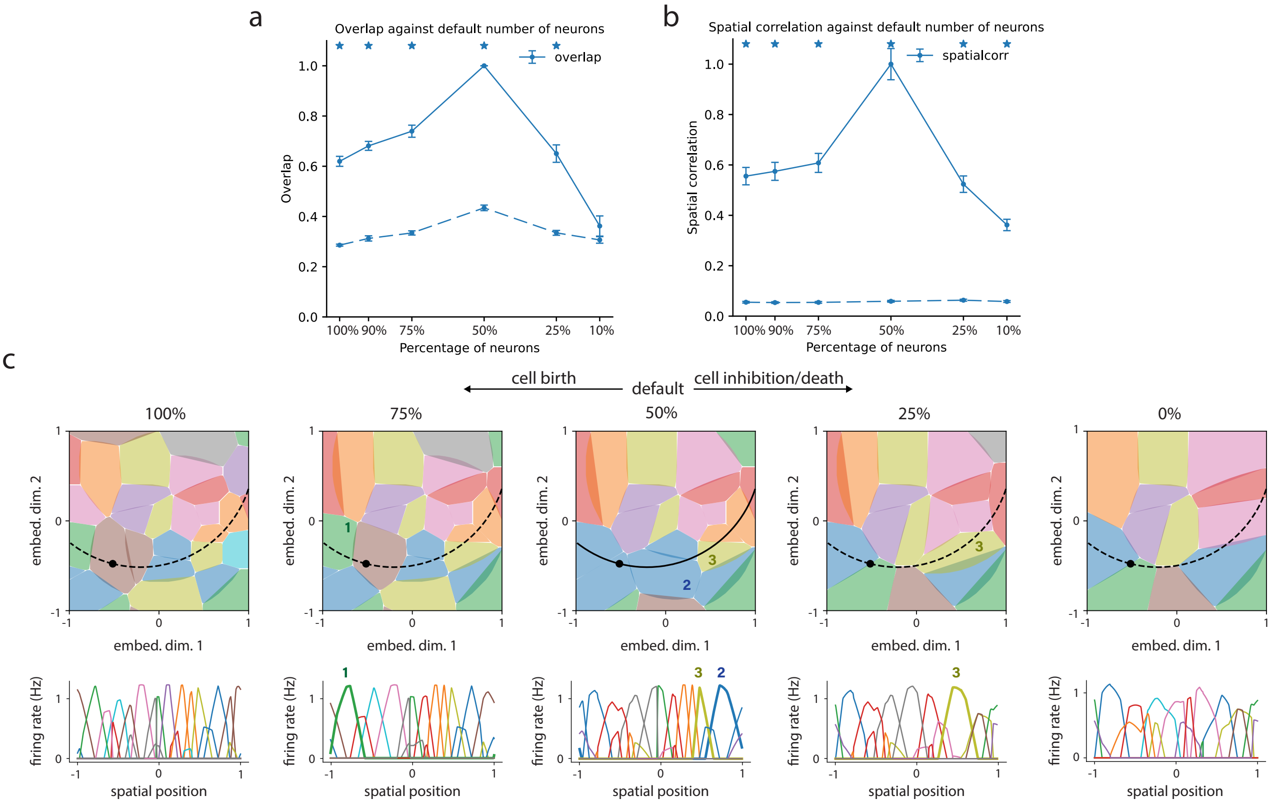

Figure S6: **Null-space remapping analysis.**
a, b: Overlap and spatial correlation (solid) along with shuffle controls (dashed), comparing "default" network (50% sparsity) to NS remapping where other amounts of sparsity are chosen (*spar*, see Methods Section 3.2.2); mean and SEM computed for 5 random selections of suppressed neurons in each case. Stars mark where the mean overlap is significantly different from the shuffle mean (t-test, Bonferroni correction with $n = 6$, see Methods Section 4). **c**: Example trajectories and place fields visualized in angle space (top) and as a function of position (bottom) for different levels of sparsity, following panels (**a, b**). Three neurons highlighted (1, 2, & 3) highlighting dropping in and out, and small tuning modulations. For all panels, note that *spar* > 50% indicates cell birth and *spar* < 50% indicates suppression or cell death.

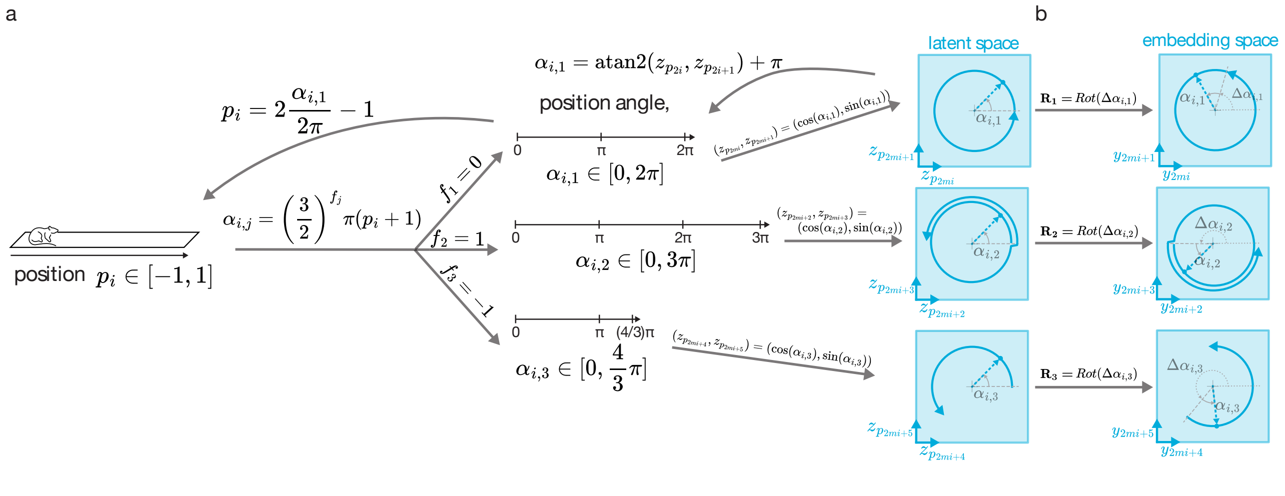

Figure S7: **Grid realignment: encoding and decoding with multiple phase-shifted modules.**
a: Exemplified encoding from a single environmental variable $p_i \in [-1, 1]$ to its latent representation $(z_{p_{2mi}}, z_{p_{2m(i+1)-1}}) \in S_1 \times \cdots \times S_1$ through angular encoding and corresponding decoding. Here using $m = 3$ modules with frequency parameters $f_1 = 0$, $f_2 = 1$ and $f_3 = -1$. Decoding is done only using the first module due to the restriction $f_1 = 0$. **b**: Exemplified embedding from this latent representation $(z_{p_{2mi}}, z_{p_{2m(i+1)-1}}) \in S_1 \times \cdots \times S_1$ to the embedding space $\mathbf{y} \in S_1 \times \cdots \times S_1$ through multiplication with a phase shift matrix $\mathbf{y} = \mathbf{R}\mathbf{z_p}$ (see Eq. 22), constructed with smaller rotation matrices $\mathbf{R}_1, \mathbf{R}_2, \mathbf{R}_3$.

bioRxiv preprint doi: https://doi.org/10.1101/2025.03.14.643251; this version posted August 11, 2025. The copyright holder for this preprint (which was not certified by peer review) is the author/funder, who has granted bioRxiv a license to display the preprint in perpetuity. It is made available under aCC-BY 4.0 International license.

	High dim. (Fig. S3)	Low dim. (Fig. 3e,f, S2a)	Low dim vis. (Fig. 3b-d)	Low dim analysis (Fig. S1, S3)	Grid cells (Fig. 3k,l, S2b)	Grid cells vis. (Fig. 3h-j)
P	2		1	2	2	1
Y	128	128	3	$2^{[4,6]} = 16, 32, 64$	12	4
$\frac{N}{Y}$	1	16	8	$2^{[0,6]} = 1, \ldots, 64$	8	8
N	128	2048	24	$2^{[4,12]} = 16, \ldots, 4096$	96	24
$\mathbf{D}$	$\mathbf{Id}_{N \times N}$	$U_{norm}(Y \times N)$			$U_{norm}(Y \times N)$	
C or M	M	M	M	M	C	C
$\mathbf{z_p}$	$m = 1$				$m = 3$, $f_j \in [0, 1, -1]$	$m = 2$, $f_j \in [0, 1]$
$\mathbf{z}$	$\mathbf{z} = \mathbf{z_p}$				$\mathbf{z} = \mathbf{z_p}$	
$\mathbf{y} = \mathbf{Rz}$	$U_{ortho}(N \times N)$	$U_{ortho}(Y \times N)$			$diag(Rot(2 \times 2))$	
envs	10		2	30	10	2

Table S1: Encoder-decoder (ED) remapping. The notation $2^{[a,b]}$ stands for all powers of 2^j with integers $j \in [a, b]$

	Space-feature (Fig. 4e,f, S2c)	Space-feature vis. (Fig. 4b-d)	Space-feature analysis (Fig. S5)	Reward vis. (Fig. 6c-f)	Implicit-space (Fig. 4k,l, S2d)	Implicit-space vis. (Fig. 4h-j)
P	2	1	2	1	2	1
Y	64	4	$2^{[3,6]} = 8, \ldots, 64$	4	64	4
$\frac{N}{Y}$	16	8	$2^{[0,4]} = 1, \ldots, 16$	4	16	8
N	1024	32	$2^{[3,10]} = 8, \ldots, 1024$	16	1024	32
$\mathbf{D}$	$U_{norm}(Y \times N)$				$U_{norm}(Y \times N)$	
C or M	CM			CM and pM	M	
$\mathbf{c}$	$U([-1, 1]^C) + GP(\mathbf{0}, K)$		$\mathcal{N}(\mathbf{0}, \sigma) + GP(\mathbf{0}, \sigma K)$, σ=0.1,0.2,0.5,1	$\text{pdf}_{\mathcal{N}(\mu_r, \sigma Id)}$, $\sigma = 0.01$	$U([-1, 1]^C) + GP(\mathbf{0}, K)$	
$\mathbf{z}$	$\mathbf{z} = (\mathbf{z_p}, \mathbf{z_c})$				$\mathbf{z} = \mathbf{z_c}$	
$\mathbf{y}$	$\mathbf{y} = \mathbf{z}$, $\lVert \mathbf{y}_{j \in P} \rVert = \lVert \mathbf{y}_{j \in C} \rVert = 1/\sqrt{2}$				$\mathbf{y} = \mathbf{z}$	
envs	10	3	30	2	10	3

Table S2: Mixed-selective (MS) remapping. The notation $2^{[a,b]}$ stands for all powers of 2^j with integers $j \in [a, b]$

bioRxiv preprint doi: https://doi.org/10.1101/2025.03.14.643251; this version posted August 11, 2025. The copyright holder for this preprint (which was not certified by peer review) is the author/funder, who has granted bioRxiv a license to display the preprint in perpetuity. It is made available under aCC-BY 4.0 International license.

	Null-space (Fig. S2e)	Null-space vis. (Fig. 5e-g)	Null-space analysis (Fig. S6)
P	2	1	2
Y	128	3	16
$\frac{N}{Y}$	32	16	16
N	4096	48	256
$\mathbf{D}$	$U_{norm}(Y \times N)$		
C or M	M		
$spar$	1		[1, 0.9, 0.75, 0.5, 0.25, 0.1]
$\mathbf{T}$	$\mathbf{T}_{active} = 10.5$		
$\mathbf{z}$	$\mathbf{z} = \mathbf{z_p}$		
$\mathbf{y} = \mathbf{Rz}$	$U_{ortho}(Y \times N)$		
envs	1	1	5

Table S3: Null-space (NS) remapping.
